# Antigen-Independent Maturation of CD2, CD11a/CD18, CD44, and CD58 Expression on Thymic Emigrants in Fetal and Postnatal Sheep

**DOI:** 10.1155/1995/35075

**Published:** 1995

**Authors:** Deborah A. Witherden, Nevin J. Abernethy, Wayne G. Kimpton, Ross N. P. Cahill

**Affiliations:** 1 Laboratory for Foetal and Neonatal Immunology The University of Melbourne Cnr. Flemington Road and Park Drive Parkville Victoria 3052 Australia; 2 IGBMC BP163 Illkirch Cedex C.U. de Strasbourg 67404 France; 3 Department of Molecular Medicine University of Auckland New Zealand

**Keywords:** Thymic emigrants, CD2, CD11a/18, CD44, CD58, fetal, *γδ* T cells

## Abstract

We have compared the expression of CD2, CD11a/CD18, CD44, and CD58 on *αβ* and *γδ* T cells emigrating from the fetal and postnatal thymus. We report that both *γδ* and the CD4^+^ CD8^-^ and CD4^-^CD8^+^ subsets of *αβ* T cells express mature levels of the adhesion molecules CD11a/CD18, CD44, and CD58 upon emigration from the thymus. Whereas CD44 is up-regulated on *γδ*^+^ thymocytes prior to export, down-regulation of both CD11a/CD18 and CD58 occurs prior to emigration from the thymus, suggesting that down-regulation of these molecules may be a final maturational step taken by developing *γδ* T cells before their export from the thymus. In contrast, there is continued up-regulation of CD2 on *αβ* and *γδ* T cells upon emigration from the thymus and as they move into the mature peripheral T-cell pool. There was also a marked reduction in the number of CD2^+^ *γδ* T cells exported during fetal development that was associated with a marked reduction in the percentage of CD2^2+^ *γδ* thymocytes exported. The postthymic maturation of CD2 and the other changes in adhesion-molecule expression appear to be independent of extrinsic antigen, as the same changes were observed in the antigen-free environment of the fetus as in the postnatal lamb, which has been exposed to extrinsic antigen. It thus appears that these changes in adhesion-molecule expression are as a result of the normal maturation pathway from a developing thymocyte to a mature peripheral T cell.


*Developmental Immunology*, 1995, Vol 4, pp. 199–209
Reprints available directly from the publisher
Photocopying permitted by license only

© 1995 Harwood Academic Publishers GmbH
Printed in Singapore

### Antigen-Independent Maturation of CD2, CD11a/CD18, CD44, and CD58 Expression on Thymic Emigrants in Fetal and Postnatal Sheep

DEBORAH A. WITHERDEN,[†] NEVIN J. ABERNETHY,[‡] WAYNE G. KIMPTON,[§] and ROSS N.P. CAHILL*[§]

*[§]Laboratory for Foetal and Neonatal Immunology, The University of Melbourne, Cnr. Flemington Road and Park Drive, Parkville, VIC, Australia 3052*
*[†]IGBMC, BP163, 67404, Illkirch Cedex, C.U. de Strasbourg, France*
*[‡]Department of Molecular Medicine, University of Auckland, New Zealand*

We have compared the expression of CD2, CD11a/CD18, CD44, and CD58 on αβ and γδ T cells emigrating from the fetal and postnatal thymus. We report that both γδ and the $CD4^+CD8^-$ and $CD4^-CD8^+$ subsets of αβ T cells express mature levels of the adhesion molecules CD11a/CD18, CD44, and CD58 upon emigration from the thymus. Whereas CD44 is up-regulated on $\gamma\delta^+$ thymocytes prior to export, down-regulation of both CD11a/CD18 and CD58 occurs prior to emigration from the thymus, suggesting that down-regulation of these molecules may be a final maturational step taken by developing γδ T cells before their export from the thymus. In contrast, there is continued up-regulation of CD2 on γδ and αβ T cells upon emigration from the thymus and as they move into the mature peripheral T-cell pool. There was also a marked reduction in the number of $CD2^+$ γδ T cells exported during fetal development that was associated with a marked reduction in the percentage of $CD^{2+}$ γδ thymocytes exported. The postthymic maturation of CD2 and the other changes in adhesion-molecule expression appear to be independent of extrinsic antigen, as the same changes were observed in the antigen-free environment of the fetus as in the postnatal lamb, which has been exposed to extrinsic antigen. It thus appears that these changes in adhesion-molecule expression are as a result of the normal maturation pathway from a developing thymocyte to a mature peripheral T cell.

KEYWORDS: Thymic emigrants, CD2, CD11a/18, CD44, CD58, fetal, γδ T cells.

### INTRODUCTION

Adhesion molecules are crucial for mature T-cell function and they have also been implicated in T-cell ontogeny and differentiation (Hogg and Landis, 1993; Janeway Jr. and Golstein, 1993). Studies on human thymocytes have suggested that interaction of the adhesion molecule CD2 with its ligand CD58 is necessary for interleukin-2-receptor expression, and hence T-cell expansion, prior to surface CD3 and TCR expression (Reinherz, 1985). In addition, studies using murine fetal thymic organ cultures and transgenic mice have demonstrated a potential role for the adhesion molecule, CD11a/CD18 in αβ T-cell differentiation (Fine and Kruisbeek, 1991) and negative selection (Carlow et al., 1992). The addition of anti-CD11a/CD18 or anti-ICAM-1 antibodies markedly impairs the progression of $CD4^-CD8^-$ cells to $CD4^+CD8^+$ (Fine and Kruisbeek, 1991) and deletion of $CD4^+CD8^+$ thymocytes can be inhibited by anti-CD11a/CD18 monoclonal antibodies and partially inhibited by anti-ICAM-1 monoclonal antibodies (Carlow et al., 1992). A role for such molecules on γδ T cells has yet to be established.

The adhesion-molecule profile of T cells has also been linked to the naive and memory status of these cells. Naive αβ T lymphocytes express low levels of adhesion molecules. Shortly after antigenic stimulation, both $CD4^+$ and $CD8^+$ subsets express increased levels of adhesion molecules, which may persist after the stimulated lymphocytes have reverted to the resting state, and possibly last for the life of the memory cell (Sanders et al., 1988;

*Corresponding author.

Cerottini and MacDonald, 1989; Shimizu et al., 1990). To date, little is known about the level of expression of adhesion molecules on $\gamma\delta$ T cells at different stages of activation or maturity.

Although it has been known for over 30 years that the thymus is responsible for the initial formation and maintenance of the peripheral T-cell pool (Miller, 1961, 1991), little is known about the expression of adhesion molecules on T cells as they leave the thymus, although it has been shown that murine recent thymic emigrants included both $CD44^+$ and $CD44^-$ cells (Kelly and Scollay, 1990). In order to understand better the regulatory mechanisms controlling adhesion-molecule expression on T cells during developmental *in vivo*, it is important to know the adhesion-molecule status of T cells immediately after their export from the thymus and preferably in situations where any changes in their expression can be isolated from the effects of antigen. Sheep provide an ideal model for these purposes. It is possible to study directly naive T cells as they leave the thymus at different stages during ontogeny and it provides an additional advantage as a model because the sheep fetus is immunologically virgin (although immunologically competent), so that adhesion-molecule expression can be examined in a situation unsullied by the effects of antigen (Pearson et al., 1976; Cahill and Trnka, 1980; Kimpton et al., 1994). Examination of $\gamma\delta$ T cells is also facilitated because sheep have an unusually high proportion of $\gamma\delta$ T cells compared with mice and humans (Hein and Dudler, 1993) and thymic export of $\gamma\delta$ T cells increases during gestation to around 40% of all emigrants at 3 months of age (Witherden et al., 1994c).

Export of T cells from the murine thymus has been studied extensively by Scollay and colleagues (Scollay et al., 1980, 1984a, 1984b; Scollay, 1982). It has been shown that in mice and other species, the thymus exports about 1% of thymocytes per day (Scollay et al., 1980; Miyasaka et al., 1988, 1990; Witherden et al., 1994c) and that there are marked changes in the export of $\alpha\beta$ and $\gamma\delta$ T cells from the thymus during fetal development and after birth (Witherden et al., 1994c). Furthermore, postthymic maturation of CD45RA and L-selectin expression on thymic emigrants, which appeared to be antigen-independent, has also been shown to occur in both fetal and postnatal animals (Witherden et al., 1994a, 1994b).

The aim of this study was to examine the expression of the adhesion molecules CD2, CD11a/CD18, CD44, and CD58 on thymic emigrants in order to compare their adhesion-molecule status with that of thymocytes and mature peripheral T cells. Thymic emigrants, like fetal sheep peripheral T cells, are indisputably naive. An examination of adhesion-molecule expression on these cells therefore provided an opportunity to establish the phenotype of naive $\gamma\delta$ T cells and to look for antigen-independent, as well as possible antigen-driven, changes in the expression of these molecules on lymphocyte subsets after emigration from the thymus.

### RESULTS

### Experimental Plan

Expression of the adhesion molecules CD2, CD11a/CD18, CD44, and CD58 on thymic emigrants was investigated in five 120-day-old fetuses, four 140-day-old fetuses, and five 3-month-old lambs. Thymocytes were labeled *in situ* by direct intrathymic injection of FITC, and those cells that had migrated out of the thymus were identified as $FITC^+$ lymphocytes in blood 1 hour after intrathymic injection. One hour was chosen as a time when we could be confident that thymic emigrants would express the same surface phenotype as they did when they left the thymus and was the earliest time point when sufficient emigrants could be obtained to allow accurate phenotyping. Thymic emigrants were then phenotyped for CD2, CD11a/CD18, CD44, and CD58 expression and, using two-color immunofluorescence staining, the expression of these adhesion molecules on emigrant T-cell subsets was examined. Single-cell suspensions of ovine thymocytes and PBL were also phenotyped, although single-positive $CD4^+$ and $CD8^+$ thymocytes were not examined.

### Expression of CD2 on Thymic Emigrants: Comparison with Thymocytes and Mature T Cells

The expression of CD2 on subsets of thymocytes, thymic emigrants, and mature T cells is shown in Figs. 1 and 2. There was a marked decline in the proportion of $\gamma\delta$ emigrants expressing CD2 during fetal development, from 44% at 120 days gestation to 14% at 140 days gestation, but no further change after birth (17%, Fig. 1). This fall in the number of $CD2^+\gamma\delta^+$ emigrants coincided with an increase in the proportion of $CD2^+$ $\gamma\delta^+$ thymocytes, indicating that there was a marked drop in the percentage of $CD2^+$ $\gamma\delta^+$

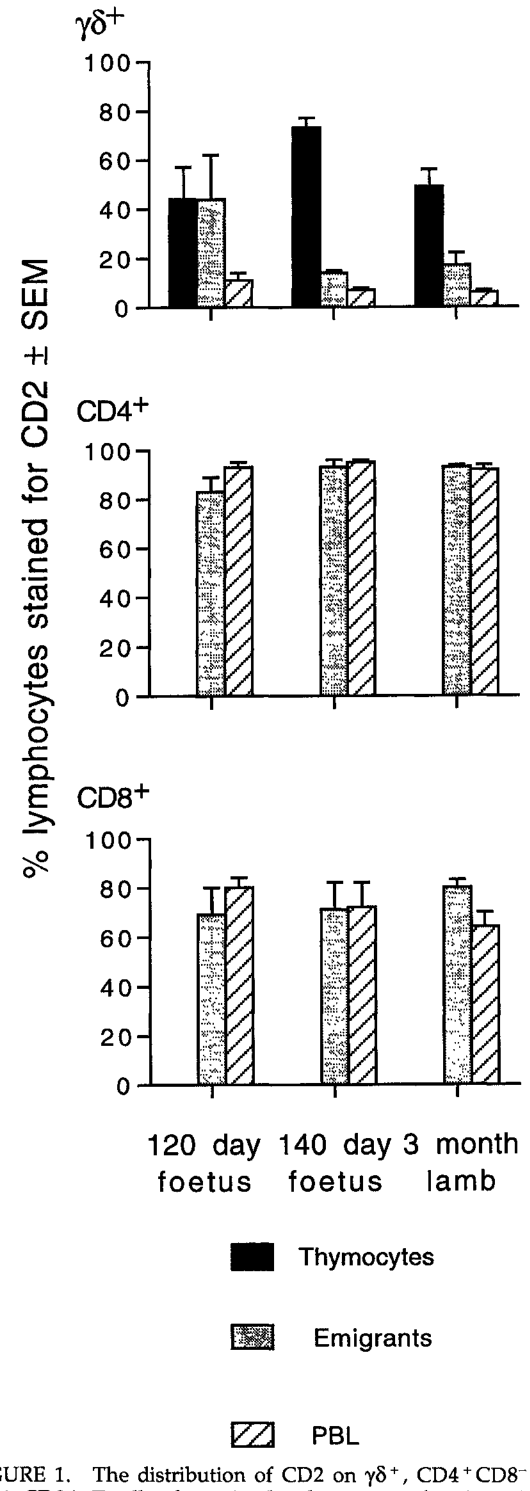

FIGURE 1. The distribution of CD2 on $\gamma\delta^+$, $CD4^+CD8^-$, and $CD4^-CD8^+$ T-cell subsets in the thymocyte, thymic emigrant, and mature peripheral blood lymphocyte populations in fetal sheep (120 and 140 days gestation) and postnatal lambs (3 months old). The proportion of $CD2^+$ thymic emigrants was determined in peripheral blood 1 hr after *in situ* labeling of the thymus with FITC. Histograms represent the mean percentages of lymphocytes stained ±SEM.

thymocytes exported from the thymus in the late-term fetus and postnatal lamb (Fig. 1). Maturation of γδ emigrants in the periphery was also associated with down-regulation of CD2 because considerably fewer γδ T cells in the mature blood population expressed CD2 than did γδ emigrants at all ages examined (Fig. 1).

In contrast to γδ emigrants, there was no difference in the percentage of $CD2^+$ αβ T-cell emigrants at different ages (Fig. 1). At all three ages, a significantly higher proportion of $CD4^+CD8^-$ and $CD4^-CD8^+$ thymic emigrants expressed CD2 than γδ emigrants. The vast majority of $CD4^+CD8^-$ cells expressed CD2 although a significant minority of around 20% of both emigrant and mature $CD4^-$ $CD8^+$ cells were $CD2^-$ (Fig. 1).

CD2 expression on αβ thymocytes was not examined; however, in contrast to $\gamma\delta^+$ cells, there was very little difference in the proportion of $CD4^+$ $CD8^-$ or $CD4^-CD8^+$ emigrants and peripheral blood lymphocytes expressing CD2 in either the fetus or the lamb (Fig. 1). The loss of CD2 between emigrants and mature T cells thus appeared to be confined to the γδ T-cell subset.

The level of CD2 expression on $\gamma\delta^+$ emigrants and peripheral blood lymphocytes is shown in Fig. 2. In all experiments, $\gamma\delta^+$ emigrants expressed CD2 at a lower level than mature $\gamma\delta^+$ peripheral blood lymphocytes (Fig. 2), but at the same level as $\gamma\delta^+$ thymocytes (data not shown). The level of CD2 expression on $CD4^+CD8^-$ and $CD4^-CD8^+$ emigrants and peripheral blood lymphocytes is also shown in Fig. 2. Like $\gamma\delta^+$ thymic emigrants, $CD4^+CD8^-$ and $CD4^-CD8^+$ emigrants also expressed a lower level of CD2 than the equivalent mature T-cell population.

### Expression of CD11a/CD18 on Thymic Emigrants: Comparison with Peripheral Blood Lymphocytes and Thymocytes

The majority (90–100%) of fetal and lamb thymocytes, thymic emigrants, and peripheral blood lymphocytes expressed CD11a/CD18 (data not shown). The level of expression of CD11a/CD18 was very similar between emigrants and peripheral blood lymphocytes for all three subsets (Fig. 2). However, a comparison of $\gamma\delta^+$ thymocytes, thymic emigrants, and mature T cells revealed that both 140-day-old fetal (data not shown) and 3-month-old lamb $\gamma\delta^+$ thymocytes expressed a considerably higher level of CD11a/CD18 than $\gamma\delta^+$ emigrants and thus a higher level of CD11a/CD18 than $\gamma\delta^+$ peripheral blood lymphocytes (Fig. 3).

Very little difference in the level of expression of CD11a/CD18 on $\alpha\beta$ and $\gamma\delta$ subsets was apparent except for the emergence of a small CD11a/CD18$^{hi}$ population amongst CD4$^-$CD8$^+$ peripheral blood lymphocytes that appeared late in gestation and was still apparent after birth (Fig. 2). This CD11a/CD18$^{hi}$ population was not present amongst the CD4$^+$CD8$^-$ or $\gamma\delta^+$ subsets or amongst any of the three subsets at 120 days gestation, nor was it detectable in any thymic emigrant population.

### Expression of CD44 and CD58 on Thymic Emigrants: Comparison with Peripheral Blood Lymphocytes and Thymocytes

The majority of CD4$^+$CD8$^-$, CD4$^-$CD8$^+$, and $\gamma\delta^+$ thymic emigrants and peripheral blood lymphocytes (85–98%) expressed CD44 (Fig. 2) and this did not change over the three ages examined (data not shown). CD44 appeared to be up-regulated on $\gamma\delta^+$ T cells prior to export because the majority of $\gamma\delta^+$ thymocytes expressed much lower levels of CD44 than $\gamma\delta^+$ emigrants or peripheral blood lymphocytes (Fig. 3). Whereas the majority of $\gamma\delta^+$ emigrants and peripheral blood lymphocytes expressed similar levels of CD44, there was an appreciable number of CD44$^{lo}$ $\gamma\delta^+$ cells in the mature blood T-cell population (Fig. 3). It was also apparent that many $\gamma\delta$ emigrants and peripheral blood lymphocytes expressed considerably lower levels of CD44 than did the corresponding CD4$^+$CD8$^-$ populations (Fig. 2).

The majority of CD4$^+$CD8$^-$, CD4$^-$CD8$^+$, and $\gamma\delta^+$ thymic emigrants and peripheral blood lymphocytes (80–100%) expressed CD58 (Fig. 2), and as with CD44 expression, this did not change over the three ages examined (data not shown). Most $\gamma\delta^+$ thymocytes expressed much higher levels of CD58

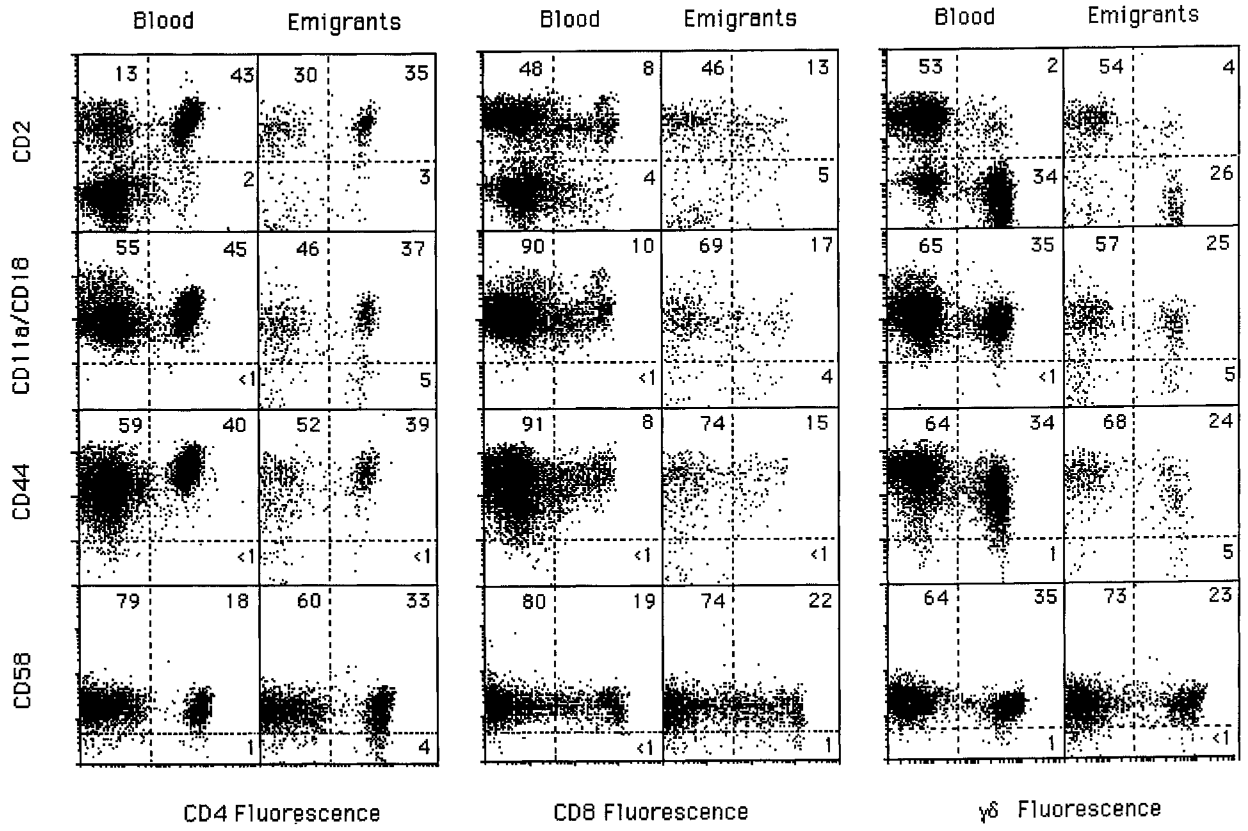

FIGURE 2. Two-color FACS plots of CD2, CD11a/CD18, CD44, and CD58 expression versus CD4, CD8, and $\gamma\delta$ expression on mature peripheral blood lymphocytes and thymic emigrants from 3-month-old lambs. Emigrants were identified by *in situ* FITC labeling, and the surface phenotypes for both mature T cells and thymic emigrants were determined by two-color immunofluorescence staining using phycoerythrin (adhesion molecules) and Texas Red (T-cell subset). Quadrant markers were set based on isotype-matched control mAbs.

than γδ⁺ thymic emigrants and mature T cells suggesting that CD58 was down-regulated on γδ⁺ T cells prior to their export from the thymus (Fig. 3). The expression of CD58 on αβ T cells in the thymus was not examined but $CD4^+CD8^-$ and $CD4^-CD8^+$ emigrants and mature T cells expressed the same low levels of CD58 as the corresponding γδ T-cell populations (Fig. 2).

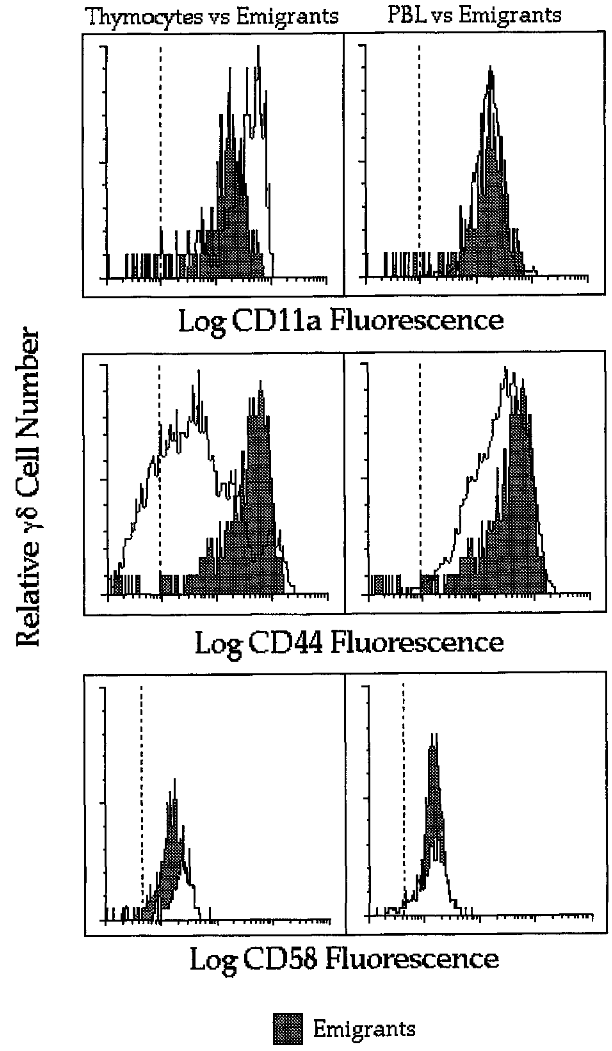

FIGURE 3. Level of expression of CD11a/CD18, CD44, and CD58 on $\gamma\delta^+$ thymocytes, thymic emigrants (shaded), and peripheral blood lymphocytes in 3-month-old lambs. Emigrants were identified by *in situ* FITC labeling, and the surface phenotypes were determined by two-color immunofluorescence staining using phycoerythrin (adhesion molecules) and Texas Red ($\gamma\delta^+$ cells). Dash lines represent the point beyond which staining was considered positive relative to isotype-matched control mAbs.

### DISCUSSION

There have been many studies on the up-regulation of adhesion molecules by αβ T cells upon activation and, together with CD45 isoform expression, changes in these molecules have been combined in an attempt to define the naive and memory status of T cells (Mackay et al., 1990; Picker and Butcher, 1992; Mackay, 1993a, 1993b; Picker et al., 1993a, 1993b). Very little is known about the expression of these molecules on γδ T cells, either in terms of naive and memory status, or activation state. Here, it has been possible to examine postthymic maturational changes in the expression of several adhesion molecules on both αβ and γδ T cells as they progress into the mature T-cell pool, in the very different environments that prevail in the lamb before and after birth (summarized in Fig. 4).

CD2 is a lymphocyte surface molecule thought to be critically involved in the adhesion and activation of αβ T cells during immune responses (Springer et al., 1987; Collins et al., 1994) and thymocyte expansion (Reinherz, 1985). Whereas the majority of both fetal and postnatal αβ thymic emigrants expressed CD2, most γδ emigrants in the experiments reported here were $CD2^-$. Although previous reports have described a lack of CD2 expression by γδ T cells (Mackay et al., 1988a; Giegerich et al., 1989), the experiments reported here demonstate a substantial population of $CD2^+$ γδ T-cell emigrants, which declined from 44% in 120-day-old fetuses to 17% after birth. Thus, there was a significant decrease in the export of $\gamma\delta^+CD2^+$ cells with gestational age. In the previous reports, a monoclonal antibody to T19, a cell-surface protein expressed exclusively on a subset of γδ T cells in ruminants (Mackay et al., 1989), was used to identify γδ T cells, but because γδ T cells comprise both $\gamma\delta^+T19^+$ and $\gamma\delta^+T19^-$ cells, the $\gamma\delta^+CD2^+$ cells may have been contained within the $T19^-$ subset and therefore missed in these earlier reports.

CD2 is also believed to play a crucial role as a coreceptor involved in inside-out signaling resulting in increased adhesion between the T lymphocyte and the antigen-bearing cell in both antigen-specific and antigen-independent lymphocyte activation (Collins et al., 1994). If CD2 functions as a coreceptor on γδ T cells in a similar fashion to αβ T cells, the presence of a large population of $CD2^-$ γδ thymic emigrants raises the possibility that $CD2^-$ $\gamma\delta^+$ T cells may represent an immature lineage of γδ T cells

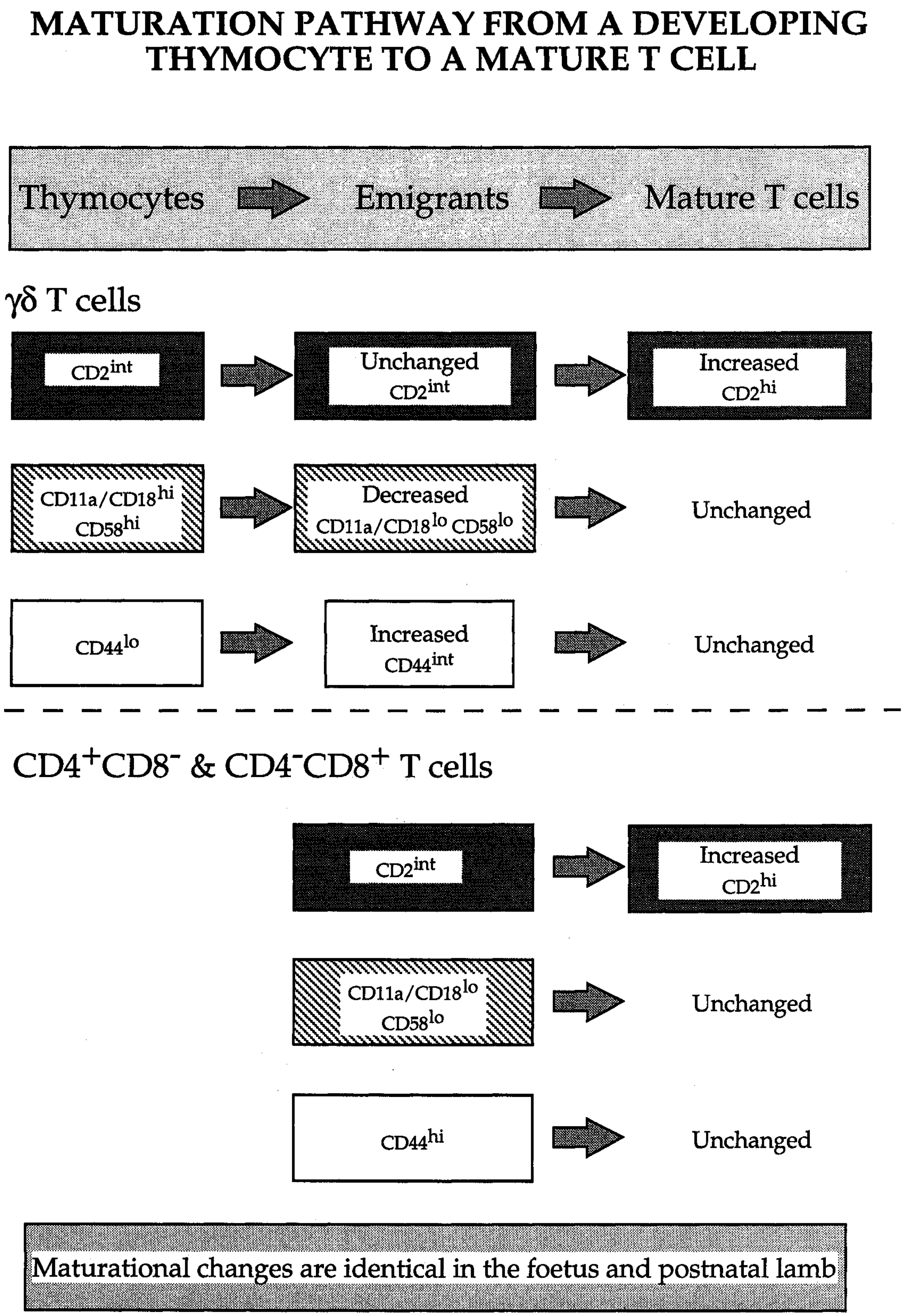

FIGURE 4. Scheme showing antigen-independent maturation of adhesion-molecule expression on γδ and αβ T cells in the fetus and postnatal lamb.

that would seem unlikely considering up to 73% of γδ T cells in the thymus express CD2 and the overwhelming majority of peripheral γδ T cells are $CD2^-$. It is also possible that $CD2^-$ and $CD2^+$ γδ T cells may represent separate lineages, as has been suggested in rats, where γδ T cells in lymph nodes are $CD2^+$, whereas those in intestinal epithelium are $CD2^-$ (Kühnlein et al., 1994). Alternatively, activation of γδ T cells may occur in a different manner from αβ T cells.

As there appeared to be developmentally regulated changes in the export of $CD2^+$ γδ T cells, we examined the level of expression of CD2 on thymic emigrants and peripheral blood lymphocytes of the fetus and lamb. In the antigen-free environment of the fetus, both thymic emigrants and mature T cells are naive cells and should therefore express the same low level of CD2. In contrast, thymic emigrants in the lamb are naive, but their mature T-cell counterparts consist of both naive and memory T cells. As such, differences in the level of expression of CD2 might be expected between thymic emigrants and peripheral blood lymphocytes in the lamb, but not in the fetus. When compared with mature peripheral blood T cells, γδ emigrants and thymocytes were found to express lower levels of CD2. This increase in CD2 expression on mature cells occurred in both fetal and postnatal lambs. Thus, the postthymic maturational changes in the expression of CD2 that occurred in the γδ T-cell population appeared to be developmentally regulated rather than due to any antigen-induced changes.

Although $CD4^+CD8^-$ and $CD4^-CD8^+$ thymocytes were not examined here, the same increased expression of CD2 was found between $CD4^+CD8^-$ and $CD4^-CD8^+$ emigrants and mature T cells, as was found for γδ T cells. In sheep, it has been reported that CD4 and CD8 single-positive medullary thymocytes, as well as a large fraction of $CD4^+CD8^-$ thymocytes, express a relatively high level of CD2, whereas the "double-negative" cells in the thymus contain mostly $CD2^-$, some $CD2^{lo}$, and a few $CD2^{hi}$ cells (Mackay et al., 1988a; Giegerich et al., 1989). This suggests that there may be down-regulation of CD2 on αβ T cells upon emigration from the thymus followed by up-regulation as these cells progress into the mature peripheral T-cell pool.

CD58 is thought to mediate intercellular adhesion of $CD58^+$ cells with thymocytes, natural killer cells, and mature αβ T lymphocytes in its role as the ligand for CD2, and its expression is up-regulated on memory cells (Springer, 1990). Along with other Ig superfamily members, CD58 can also act as a costimulatory molecule for IL-2 production and the proliferation of $CD4^+$ T cells (Hogg and Landis, 1993). An analysis of the expression of CD58 on thymic emigrants revealed that, unlike CD2, the vast majority of both γδ and αβ emigrants expressed CD58, albeit at a low level. In contrast, virtually all thymocytes expressed higher levels of CD58, suggesting that down-regulation of CD58 may be a final maturational stage of both γδ and αβ thymocytes prior to emigrating from the thymus.

CD11a/CD18 or LFA-1 is a member of the integrin family of cell-surface heterodimers that participate in a range of cell-to-cell and cell-to-extracellular matrix interactions in the immune system (Hynes, 1992). In the experiments reported here, we have shown that γδ thymocytes, in the fetus close to term and in the postnatal lamb, expressed a higher level of CD11a/CD18 than both γδ emigrants and mature T cells. Recent studies have reported that CD11a/CD18 and ICAM-1 interactions within the thymus are involved in T-cell differentiation and negative selection (Fine and Kruisbeek, 1991; Carlow et al., 1992, #1453). In this context, the down-regulation of CD11a/CD18 on γδ thymocytes may indicate a final stage in T-cell development prior to their export to the periphery.

LFA-1/ICAM-1 adhesion pathways are important in many cell-to-cell interactions in the immune system and a range of cell types such as thymocytes, peripheral blood lymphocytes, monocytes, and neutrophils have been shown to express differential levels of CD11a and CD18 (Tamatani et al., 1991a). Although we have shown there is no difference in the level of CD11a/CD18 expression on thymic emigrants and mature peripheral blood T cells, recent studies have shown that activation of lymphocytes induces qualitative changes in the avidity and ligand specificity of LFA-1 (Tamatani et al., 1991b). For example, qualitative but not quantitative changes in the expression of LFA-1 on lymphocytes result in the binding of activated, but not resting lymphocytes, to high endothelial venules in lymph nodes (Tamatani et al., 1991b).

Very little difference was apparent in the expression of CD11a/CD18 between the mature T-cell subsets with one exception. At 140 days gestation and 3 months of age, a small population of $CD4^-CD8^+$ lymphocytes expressed a high level of CD11a/CD18. This $CD11a/CD18^{hi}$ population represented a greater proportion in the 3-month-old lamb than in the fetus at term. As this population was not present in the young fetus, but was present in the fetus at 140 days gestation, it cannot be due to antigen-induced activation of the $CD4^-CD8^+$ cells, as the ovine fetus is devoid of extrinsic antigen.

CD44 is a polymorphic cell-surface protein that is expressed on a wide variety of cell types and is believed to be involved in a variety of biological reactions by recognizing multiple ligands (Haynes et al., 1989; Toyama-Sorimachi et al., 1993; Toyama-Sorimachi and Miyasaka, 1994). In humans, CD44 is expressed at uniformly low levels on naive T cells (Picker et al., 1990), whereas memory T cells in both mice and humans have been shown to express higher levels of CD44 than their naive counterparts (Budd et al., 1987; Sanders et al., 1988). In the experiments reported here, fetal and postnatal αβ and γδ emigrants expressed CD44 at the same level as mature T cells, suggesting that CD44 expression is not easily related to naive or memory status. Kelly and Scollay (1990) have shown that a proportion of thymic emigrants and mature T cells in both 5-day-old and adult mice express CD44, indicating that CD44 is also expressed on virgin T cells in mice. In our experiments, a population of $CD44^{lo}$ γδ T-cell emigrants that emerged in fetal life was also present in lambs in the mature T-cell population, suggesting developmental rather than antigenic regulation. In contrast, the experiments of Kelly and Scollay (1990) indicated a clear increase in CD44 expression from thymic emigrants to mature T cells, so that either different isoforms of CD44 were being detected in these studies or there is a major difference in the expression of CD44 between the two species.

An interesting observation was that emigrants expressed higher levels of CD44 than the majority of thymocytes, suggesting up-regulation of CD44 before emigration. In addition, there was a small population of thymocytes that expressed the same high levels of CD44 as thymic emigrants, possibly representing mature thymocytes that are about to emigrate. This supports there being selective export of $CD44^{hi}$ cells from the ovine thymus, as has been suggested for the human and inferred for the sheep, by the high-level expression of CD44 on human and ovine medullary thymocytes (de los Toyos et al., 1989), a situation quite different from that in the mouse.

Nothing is known of the factors that regulate cell exit from the thymus or of any role for adhesion molecules in the exit mechanism(s). The vast majority of γδ T cells express the adhesion molecules CD11a/CD18, CD44, and CD58, but only a proportion express the adhesion molecule CD2, both as thymic emigrants and mature T cells. Whereas it is unclear whether the down-regulation of CD11a/CD18 and CD58 on γδ T cells is necessary before γδ T cells can leave the thymus, it is clear that the expression of CD2 is not mandatory for γδ T-cell export. Upon emigration from the thymus, both γδ and αβ T cells express mature levels of CD44, CD11a/CD18, and CD58, but there is postthymic maturation of CD2 expression. The changes in adhesion-molecule expression on thymic emigrants reported here, and the changes in L-selectin and CD45RA expression on thymic emigrants (Witherden et al., 1984a, 1994b) previously reported apparently do not rely on the presence of antigen, but would seem to occur as part of the normal development of a T cell.

### MATERIALS AND METHODS

### Animals

Fetal lambs of known gestational age were obtained from timed matings of virgin merino ewes with merino rams, 120 and 140 days prior to the experiments. Three-month-old postnatal lambs were obtained from the same breeding program.

### Intrathymic Injection of FITC

For injection of FITC into the fetal thymus, the uterus was exteriorized and the fetus exposed according to the procedure described previously (Smeaton, et al., 1969). The head and neck of the fetus were then delivered through an incision in the uterus. From this point, the procedures for exposing the thymus and the intrathymic injection of FITC were essentially the same for both fetal and postnatal animals. A midline incision was made in the neck of the fetus or the lamb and the cervical thymus exposed after blunt dissection of the overlying tissue. A small biopsy of thymus was taken for phenotypic analysis. An aqueous solution of FITC, at 500 μg/ml, was then heated to 37°C and injected at multiple sites directly into the thymus, essentially according to the technique described previously for the adult murine thymus (Weissman, 1967; Scollay et al., 1980). Blood was taken 1 h following intrathymic injection for phenotypic analysis. Blood samples were taken from the jugular vein, carotid artery, or by cardiac puncture, and heparin was added to a final concentration of 100 U/ml (Commonwealth Serum Laboratories, Parkville, Victoria, Australia). Red cells were lysed using $NH_4Cl$ red cell lysis buffer (Mackay et al., 1988b).

### Monoclonal Antibodies

Monoclonal antibodies (mAb) directed against the lymphocyte cell-surface antigens; CD4 (44-38/44-97), CD8 (38-65), the $\gamma\delta$ TCR (86D), CD2 (36F), CD11a/CD18 (F10-150), CD44 (25-32), and CD58 (L180-1) were obtained as undiluted tissue-culture supernatant or as ascites fluid from Dr. M. Brandon (University of Melbourne, Victoria, Australia) and Dr. C. Mackay (Basel Institute for Immunology, Basel, Switzerland) and have been described previously (Hünig, 1985; Maddox et al., 1985; Mackay et al., 1988a; 1988c; 1989; 1990; 1992). For two colour immunofluorescence staining, the mAb to CD4, CD8, and the $\gamma\delta$ TCR were used as purified proteins conjugated to biotin (Goding, 1986).

### Thymocyte Suspensions

The cervical thymus was removed and placed into HBSS (ICN-Biomedicals, Seven Hills, NSW, Australia) containing 2% lamb serum (Gibco Laboratories, NY) (HBSS-2%LS) at 4°C. Suspensions were made by teasing the organs through a wire mesh into HBSS-2%LS. The cells were washed three times by centrifugation (10 min, 300 g, swing-out rotor, 4°C) and resuspended in HBSS-2%LS.

### Immunofluorescence Staining and Flow Cytometry

All reagents were pretitrated and used at optimum concentrations to give maximal positive staining with minimal background. Before immunofluorescence staining and between reaction steps, cells were washed three times in HBSS-2%LS. All incubations were for 30 min and cells were kept on ice or at 4°C at all times. $10^7$ lymphocytes were first incubated with 50 μl mAb, followed by 50 μl phycoerythrin (PE) conjugated anti-mouse Ig $F(ab')_2$ (Silenus Laboratories, Hawthorn, Victoria, Australia). In order to saturate free-binding sites on the anti-mouse Ig, cells were incubated with a 10-fold excess of mouse Ig (w/w compared to the anti-mouse Ig; Chemicon International Inc., CA). Cells were then incubated with 50 μl biotinylated mAb followed by 50 μl of a streptavidin-conjugated tandem of PE and Texas red (Tandem, Southern Biotechnology, Birmingham, AL). Cells were fixed in 100 μl ($10^8$ cells/ml) of 3% paraformaldehyde and kept in the dark at 4°C for no longer than a week before FACS analysis. Flow cytometric analysis was performed using a FACScan (Becton Dickinson, Sunnyvale, CA). Thymocytes and peripheral blood lymphocytes were identified by forward angle and 90° light scatter. Phenotypic analysis of $FITC^+$ cells (thymic emigrants) was performed by triggering on FITC rather than on forward scatter and electronic gating on forward angle and 90° light scatter, allowing more efficient data collection. Nonspecific staining was determined by an isotype-matched control mAb. Comparisons of fluorescence intensity were only made between samples that were stained and analyzed at the same time, and care was taken to ensure they contained similar numbers of positive cells. Except for thymic emigrants ($FITC^+$ cells), where as many cells as possible were analyzed (approximately 1000), 10,000 cells were analyzed for each sample.

### Statistical Analysis

The percentage of each subset was compared between age groups and between lymphocyte sources using a one-way analysis of variance. Those showing variation were then compared using an unpaired Student's *t*-test (Glantz, 1987). A value of $p \leq 0.05$ was considered significant.

### ACKNOWLEDGMENTS

We would like to thank Ms. J.E. Holder and Dr. E.A. Washington for their help in preparing this manuscript. This work was supported by the National Health and Medical Research Council of Australia.

*(Received October 20, 1995)*

*(Accepted May 5, 1995)*

### REFERENCES

Budd R.C., Cerottini J., Horvath C., Bron C., Pedrazzini T., Howe R.C., and MacDonald H.R. (1987). Distinction of virgin and memory T lymphocytes. Stable acquisition of the Pgp-1 gly coprotein concomitant with antigenic stimulation. J. Immunol. 13: 3120–3129.

Cahill R.N.P., and Trnka Z. (1980). Growth and development of recirculating lymphocytes in the sheep fetus. In: Essays on the anatomy and physiology of lymphoid tissues, Trnka Z, and Cahill R.N.P., Eds. (Basel: Karger), pp. 38–49.

Carlow D.A., van Oers N.S., Teh S.-J., and Teh H.-S. (1992). Deletion of antigen-specific immature thymocytes by dendritic cells requires LFA-1/ICAM interactions. J. Immunol. 148: 1595–1603.

Cerottini J., and MacDonald H.R. (1989). The cellular basis of T-cell memory. Ann. Rev. Immunol. 7: 77–89.

Collins T.L., Kassner P.D., Bierer B.E., and Burakoff S.J. (1994). Adhesion receptors in lymphocyte activation. Curr. Opin. Immunol. 6: 385–393.

de los Toyos J., Jalkanen S., and Butcher E.C. (1989). Flow cytometric analysis of the Hermes homing-associated antigen on human lymphocyte subsets. Blood 74: 751–760.

Fine J.S., and Kruisbeek A.M. (1991). The role of LFA-1/ICAM-1 interactions during murine T lymphocyte development. J. Immunol. 147: 2852–2859.

Giegerich G.W., Hein W.R., Miyasaka M., Tiefenthaler G., and Hünig T. (1989). Restricted expression of CD2 among subsets of sheep thymocytes and T lymphocytes. Immunol. 66: 354–361

Glantz S.A. (1987). Primer of biostatistics (New York: McGraw-Hill), p. 291.

Goding J.W. (1986). Monoclonal antibodies: Principles and practice: Production and application of monoclonal antibodies in cell biology, biochemistry and immunology (London: Academic Press).

Haynes B.F., Telen M.J., Hale L.P., and Denning S.M. (1989). CD44—A molecule involved in leukocyte adherence and T-cell activation. Immunol. Today 10: 423–428.

Hein W.R., and Dudler L. (1993). Divergent evolution of T cell repertoires: Extensive diversity and developmentally regulated expression of the sheep γδ T cell receptor. EMBO J. 12: 715–724.

Hogg N., and Landis R.C. (1993). Adhesion molecules in cell interactions. Curr. Op. Immunol. 5: 383–390.

Hünig T. (1985). The cell surface molecule recognized by the erythrocyte receptor of T lymphocytes. J. Exp. Med. 162: 890–901.

Hynes R.O. (1992). Integrins: Versitility, modulation and signalling in cell adhesion. Cell 69: 11–15.

Janeway Jr C.A., and Golstein P. (1993). Lymphocyte activation and effector functions. Editorial review: The role of cell surface molecules. Curr. Op. Immunol. 5: 313–323.

Kelly K.A., and Scollay R. (1990). Analysis of recent thymic emigrants with subset- and maturity-related markers. Int. Immunol. 2: 419–425.

Kimpton W.G., Washington E.A., and Cahill R.N.P. (1994). The development of the immune system in the foetus. In: Textbook of foetal physiology, Thorburn G.D., and Harding R., Eds. (Oxford: Oxford University Press), pp. 245–255.

Kühnlein P., Park J., Herrmann T., Elbe A., and Hünig T. (1994). Identification and characterization of rat γ/δ T lymphocytes in peripheral lymphoid organs, small intestine, and skin with a monoclonal antibody to a constant determinant of the γ/δ T cell receptor. J. Immunol. 153: 979–986.

Mackay C.R. (1993a). Homing of naive, memory and effector lymphocytes. Curr. Op. Immunol. 5: 423–427.

Mackay C.R. (1993b). Immunological memory. Adv. Immunol. 53: 217–265.

Mackay C.R., Beya M.-F., and Matzinger P. (1989). γ/δ T cells express a unique surface molecule appearing late during thymic development. Eur. J. Immunol. 19: 1477–1483.

Mackay C.R., Hein W.R., Brown M.H., and Matzinger P. (1988a). Unusual expression of CD2 in sheep: Implications for T cell interactions. Eur. J. Immunol. 18: 1681–1688.

Mackay C.R., Kimpton W.G., Brandon M.R., and Cahill R.N.P. (1988b). Lymphocyte subsets show marked differences in their distribution between blood and the afferent and efferent lymph of peripheral lymph nodes. J. Exp. Med. 167: 1755–1765.

Mackay C.R., Maddox J.F., Wijffels G.L., Mackay I.R., and Walker I.D. (1988c). Characterization of a 95,000 molecule on sheep leukocytes homologous to murine Pgp-1 and human CD44. Immunology 65: 93–99.

Mackay C.R., Marston W.L., and Dudler L. (1990). Naive and memory T cells show distinct pathways of lymphocyte recirculation. J. Exp. Med. 171: 801–817.

Mackay C.R., Marston W.L., Dudler L., Spertini O., Tedder T.F., and Hein W.R. (1992). Tissue-specific migration pathways by phenotypically distinct subpopulations of memory T cells. Eur. J. Immunol. 22: 887–895.

Maddox J.F., Mackay C.R., and Brandon M.R. (1985). Surface antigens, SBU-T4 and SBU-T8, of sheep T lymphocyte subsets defined by monoclonal antibodies. Immunology 55: 739–748.

Miller J.F.A.P. (1961). Immunological function of the thymus. *Lancet* 2: 748–749.

Miller J.F.A.P. (1991). The discoverys of the immunological function of the thymus. Immunol. Today 12: 42–45.

Miyasaka M., Pabst R., Dudler L., Cooper M., and Yamaguchi K. (1990). Characterization of lymphatic and venous emigrants from the thymus. Thymus 16: 29–43.

Miyasaka M., Pabst R., Yamaguchiu K., and Colombo V. (1988). Lymphocyte emigration from the ovine thymus: Characterization of lymphatic emigrants and venous emigrants. In: Histophysiology of the immune system, Fossum S., and Rolstad B., Eds. (New York: Plenum), pp. 559–563.

Pearson L.D., Simpson-Morgan M.W., and Morris B. (1976). Lymphopoiesis and lymphocyte recirculation in the sheep fetus. J. Exp. Med. 143: 167–186.

Picker L.J., and Butcher E.C. (1992). Physiological and molecular mechanisms of lymphocyte homing. Ann. Rev. Immunol. 10: 561–591.

Picker L.J., Terstappen L.W.M.M., Rott L.S., Streeter P.R., Stein H., and Butcher E.C. (1990). Differential expression of homing-associated adhesion molecules by T cell subsets in man. J. Immunol. 145: 3247–3255.

Picker L.J., Treer J.R., Ferguson-Darnell B., Collins P.A., Buck D., and Terstappen L.W.M.M. (1993a). Control of lymphocyte recirculation in man. I. Differential regulation of the peripheral lymph node homing receptor L-selectin on T cells during the virgin to memory cell transition. J. Immunol. 150: 1105–1121.

Picker L.J., Treer J.R., Ferguson-Darnell B., Collins P.A., Buck D., and Terstappen L.W.M.M. (1993b). Control of lymphocyte recirculation in man. II. Differential regulation of the cutaneous lymphocyte-associated antigen, a tissue-selective homing receptor for skin-homing T cells. *J. Immunol.* 150: 1122–1136.

Reinherz E.L. (1985). A molecular basis for thymic selection: Regulation of T11 induced thymocyte expansion by the T3-Ti antigen/MHC receptor pathway. Immunol. Today 6: 75–79.

Sanders M.E., Makgoba M.W., Sharrow S.O., Stephany D., Springer T.A., Young H.A., and Shaw S. (1988). Human memory T lymphocytes express increased levels of three cell adhesion molecules (LFA-3, CD2 and LFA-1) and three other molecules (UCHL1, CDw29 and Pgp-1) and have enhanced IFN-γ production. *J. Immunol.* 140: 1410–1407.

Scollay R. (1982). Thymus cell migration: Cell migrating from thymus to peripheral lymphoid organs have a "mature" phenotype. J. Immunol. 128: 1566–1570.

Scollay R., Butcher E., and Weissman I. (1980). Thymus cell migration: Quantitative studies on the rate of migration of cells from the thymus to the periphery in mice. Eur. J. Immunol. 10: 210–218.

Scollay R., Chen W.F., and Shortman K. (1984a). The functional capabilities of cells leaving the thymus. J. Immunol. **132**: 25–30.

Scollay R., Wilson A., and Shortman K. (1984b). Thymus cell migration: Analysis of thymus emigrants with markers that distinguish medullary thymocytes from peripheral T cells. J. Immunol. **132**: 1089–1094.

Shimizu Y., van Seventer G.A., Horgan K.J., and Shaw S. (1990). Roles of adhesion molecules in T-cell recognition: Fundamental similarities between four integrins on resting human T cells (LFA-1, VLA-4, VLA-5, VLA-6) in expression, binding, and costimulation. Immunol. Rev. **114**: 109–143.

Smeaton T.C., Cole G.J., Simpson-Morgan M.W., and Morris B. (1969). Techniques for the long-term collection of lymph from the unanaesthetised foetal lamb in utero. Aust. J. Exp. Biol. Med. Sci. **47**: 565–572.

Springer T.A. (1990). Adhesion receptors of the immune system. Nature **346**: 425–434.

Springer T.A., Dustin M.L., Kishimoto T.K., and Marlin S.D. (1987). The lymphocyte function-associated LFA-1, CD2 and LFA-3 molecules: Cell adhesion receptors of the immune system. Ann. Rev. Immunol. **5**: 223–252.

Tamatani T., Kotani M., and Miyasaka M. (1991a). Characterization of the rat leukocyte integrin, CD11/CD18, by the use of LFA-1 subunit-specific monoclonal antibodies. Eur. J. Immunol. **21**: 627–633.

Tamatani T., Kotani M., Tanaka T., and Miyasaka M. (1991b). Molecular mechanisms underlying lymphocyte recirculation. II. Differential regulation of LFA-1 in the interaction between lymphocytes and high endothelial cells. Eur. J. Immunol. **21**: 855–858.

Toyama-Sorimachi N., Miyake K., and Miyasaka M. (1993). Activation of CD44 induces ICAM-1/LFA-1-independent, $Ca^{2+}$, $Mg^{2+}$-independent adhesion pathway in lymphocyte-endothelial cell interaction. Eur. J. Immunol. **23**: 439–446.

Toyama-Sorimachi N., and Miyasaka M. (1994). A novel ligand for CD44 is sulfated proteoglycan. Int. Immunol. **6**: 655–660.

Weissman I.L. (1967). Thymus cell migration. J. Exp. Med. **126**: 291–304.

Witherden D.A., Abernethy N.J., Kimpton W.G., and Cahill R.N.P. (1994a). CD45R expression of γδ and αβ T cells emigrating from the fetal and postnatal thymus. Eur. J. Immunol. **24**: 186–190.

Witherden D.A., Abernethy N.J., Kimpton W.G., and Cahill R.N.P. (1994b). Changes in thymic export of L-selectin$^{+}$ γδ and αβ T cells during fetal and postnatal development. Eur. J. Immunol. **24**: 1234–1239.

Witherden D.A., Kimpton W.G., Abernethy N.J., and Cahill R.N.P. (1994c). Changes in thymic export of γδ and αβ T cells during fetal and postnatal development. Eur. J. Immunol. **24**: 2329–2336.

